# Bis{μ_3_-*cis*-*N*-(2-carboxyl­ato-5-chloro­phen­yl)-*N*′-[3-(dimethyl­amino)­prop­yl]oxamidato(3−)}bis­(perchlorato-κ*O*)bis­(*N*,*N*,*N*′,*N*′-tetra­methyl­ethylene­diamine)­tetra­copper(II)

**DOI:** 10.1107/S1600536811014978

**Published:** 2011-04-29

**Authors:** Yanlong Sun, Xuelian Xu

**Affiliations:** a Marine Drug and Food Institute, Ocean University of China, Qingdao 266003, People’s Republic of China

## Abstract

The title complex, [Cu_4_(C_14_H_15_ClN_3_O_4_)_2_(ClO_4_)_2_(C_6_H_16_N_2_)_2_], is a tetra­nuclear copper(II) complex lying about an inversion center wherein a *cis*-oxamide group is coordinated to both Cu atoms with bite angles of 84.45 (6) and 84.08 (10)°. Both Cu atoms adopt distorted square-pyramidal coordination geometries. The apical position of one Cu atom is occupied by an O atom from a perchlorate group, with a Cu—O bond length of 2.519 (7) Å, while the apical site of the other Cu atom is occupied by a carboxyl­ate O atom with a Cu—O distance of 2.281 (3) Å. The Cu atoms bridged by oxamide and carboxyl­ate-group bridges are separated by 5.204 (6) and 5.603 (2) Å, respectively. The crystal structure is consolidated by weak inter­molecular C—H⋯O inter­actions. Two perchlorate O atoms are disordered with unequal site-occupancy factors.

## Related literature

For the preparation of the Na[Cu(oxbm)] ligand, see: Tao *et al.* (2003 ▶). For a related crystal structure, see: Zang *et al.* (2003 ▶).
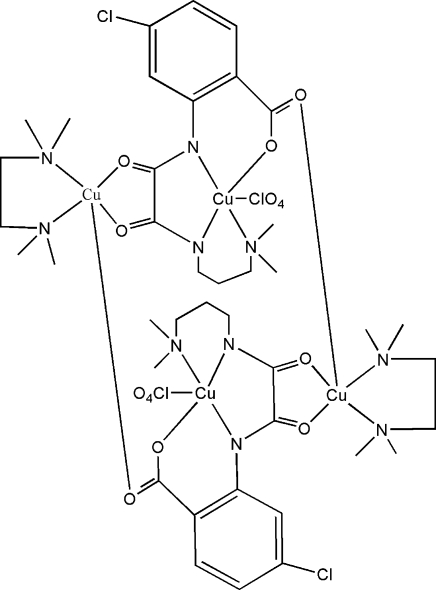


## Experimental

### 

#### Crystal data

[Cu_4_(C_14_H_15_ClN_3_O_4_)_2_(ClO_4_)_2_(C_6_H_16_N_2_)_2_]
*M* *_r_* = 1334.96
Monoclinic, 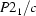

*a* = 12.5750 (13) Å
*b* = 16.4137 (19) Å
*c* = 14.1080 (15) Å
β = 113.988 (2)°
*V* = 2660.4 (5) Å^3^
*Z* = 2
Mo *K*α radiation
μ = 1.85 mm^−1^
*T* = 298 K
0.49 × 0.48 × 0.20 mm

#### Data collection

Bruker SMART CCD area-detector diffractometer
Absorption correction: multi-scan (*SADABS*; Sheldrick, 1996 ▶) *T*_min_ = 0.464, *T*_max_ = 0.708
12993 measured reflections
4682 independent reflections
3287 reflections with *I* > 2σ(*I*)
*R*_int_ = 0.052

#### Refinement

*R*[*F* ^2^ > 2σ(*F* ^2^)] = 0.044
*wR*(*F* ^2^) = 0.133
*S* = 1.00
4682 reflections
359 parameters
H-atom parameters constrained
Δρ_max_ = 0.65 e Å^−3^
Δρ_min_ = −0.76 e Å^−3^

### 

Data collection: *SMART* (Bruker, 1998 ▶); cell refinement: *SAINT* (Bruker, 1998 ▶); data reduction: *SAINT*; program(s) used to solve structure: *SHELXS97* (Sheldrick, 2008 ▶); program(s) used to refine structure: *SHELXL97* (Sheldrick, 2008 ▶); molecular graphics: *SHELXTL* (Sheldrick, 2008 ▶); software used to prepare material for publication: *SHELXL97* and *PLATON* (Spek, 2009 ▶).

## Supplementary Material

Crystal structure: contains datablocks I, global. DOI: 10.1107/S1600536811014978/pv2408sup1.cif

Structure factors: contains datablocks I. DOI: 10.1107/S1600536811014978/pv2408Isup2.hkl

Additional supplementary materials:  crystallographic information; 3D view; checkCIF report

## Figures and Tables

**Table 1:** Hydrogen-bond geometry (Å, °)

*D*—H⋯*A*	*D*—H	H⋯*A*	*D*⋯*A*	*D*—H⋯*A*
C6—H6⋯O7^i^	0.93	2.58	3.26 (3)	129
C16—H16*A*⋯O8^ii^	0.97	2.46	3.41 (3)	168
C20—H20*C*⋯O2^iii^	0.96	2.54	3.139 (6)	121

Symmetry codes: (i) 
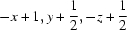
; (ii) 
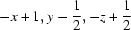
; (iii) 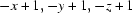.

**Fig. 1.**
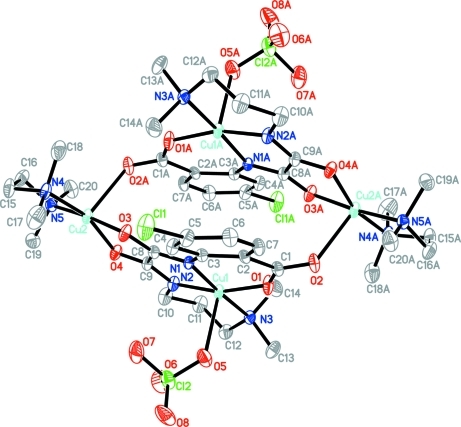
The molecular structure of (I) with 30% displacement ellipsoids (H atoms omitted for clarity). The symmetry code "A" in the atomic labels: -x+1, -y+1, -z+1.

## supplementary crystallographic information

### Comment

The title compound, (Fig. 1), is a tetranuclear copper(II) complex lying about inversion centers wherein cis-oxamido group is coordinated to Cu1 and Cu2 in an usual mode with the bite angles of 84.45 (6) and 84.08 (10)°, respectively. Both Cu1 and Cu2 atoms adopt distorted square-pyramidal coordination geometries. The basal plane around Cu1 is defined by N1, N2, N3 and O1, with maximum displacement of 0.1050 (11) Å for N1 and the Cu1 lies 0.1298 (12) Å out of this plane. The apical position of Cu1 is occupied by O5 with Cu1—O5 bond length 2.519 (7) Å. Cu2 atom is coordinated by exo-cis oxygen atoms of the oxamido ligand (O3 and O4) and the nitrogen atoms (N4 and N5) of tetramethylethylenediamine ligand thus completing the basal plane, from which the maximum deviation of an atom (O4) being 0.1233 (8) Å; Cu2 is displaced from this basal plane by 0.1835 (6) Å. The apical site of Cu2 is occupied by a carboxyl oxygen atom (O2^i^ where ^i^ = -x+1, -y+1, -z+1) with Cu2—O2^i^ distance 2.281 (3) Å. The Cu—N bond lenghts in the title complex lie in the range 1.953 (3)-2.066 (3)Å and are close to the corresponding bond lenghts reported in a copper complex (Zang *et al.*, 2003). The crystal structure is consolidated by weak intermolecular interactions of type C—H···O (Table 1).

### Experimental

The Na[Cu(oxbm)] ligand, Na[Cu(oxamido-N-[3-N,N'-dimethylaminopropyl]-N'-(4-Chloro) -benzoato)], was prepared according to Tao *et al.*, (2003). A methanol (10 ml) solution of Cu(ClO_4_)_2_^.^6H_2_O (0.742 g, 2 mmol) was added dropwise into a water solution (10 ml) of  Na[Cu(oxbm)] (0.854 g, 2 mmol) with continuous stirring. The mixture was stirred for an hour and then tetramethylethylenediamine (0.0224 g, 2 mmol) in methanol (10 ml) was added dropwise. The solution obtained was stirred at 333 K for 10 h. The resulting solution was then filtered and the filtrate was allowed to stand at room temperature for three weeks to give well shaped green crystals of the title complex suitable for X-ray analysis.

### Refinement

H atoms were positioned geometrically [0.93 (CH), 0.97 (CH_2_) and 0.96 (CH_3_)Å] and constrained to ride on their parent atoms with *U*_iso_(H) =1.2(1.5 for methyl)*U*_eq_(C). Two O-atoms of the perchlorate were disordered and their site occupancy factors were determined at earlier stages of refinement and were fixed at these occupancy factors during the final stages of refinements.

### Crystal data

**Table:**

[Cu_4_(C_14_H_15_ClN_3_O_4_)_2_(ClO_4_)_2_(C_6_H_16_N_2_)_2_]	*F*(000) = 1368
*M**_r_* = 1334.96	*D*_x_ = 1.666 Mg m^−^^3^
Monoclinic, *P*2_1_/*c*	Mo *K*α radiation, λ = 0.71073 Å
Hall symbol: -P 2ybc	Cell parameters from 4604 reflections
*a* = 12.5750 (13) Å	θ = 2.2–27.7°
*b* = 16.4137 (19) Å	µ = 1.85 mm^−^^1^
*c* = 14.1080 (15) Å	*T* = 298 K
β = 113.988 (2)°	Block, green
*V* = 2660.4 (5) Å^3^	0.49 × 0.48 × 0.20 mm
*Z* = 2	

### Data collection

**Table:**

Bruker SMART CCD area-detector diffractometer	4682 independent reflections
Radiation source: fine-focus sealed tube	3287 reflections with *I* > 2σ(*I*)
graphite	*R*_int_ = 0.052
φ and ω scans	θ_max_ = 25.0°, θ_min_ = 1.8°
Absorption correction: multi-scan (*SADABS*; Sheldrick, 1996)	*h* = −7→14
*T*_min_ = 0.464, *T*_max_ = 0.708	*k* = −19→19
12993 measured reflections	*l* = −16→14

### Refinement

**Table:**

Refinement on *F*^2^	Primary atom site location: structure-invariant direct methods
Least-squares matrix: full	Secondary atom site location: difference Fourier map
*R*[*F*^2^ > 2σ(*F*^2^)] = 0.044	Hydrogen site location: inferred from neighbouring sites
*wR*(*F*^2^) = 0.133	H-atom parameters constrained
*S* = 1.00	*w* = 1/[σ^2^(*F*_o_^2^) + (0.072*P*)^2^ + 1.0008*P*] where *P* = (*F*_o_^2^ + 2*F*_c_^2^)/3
4682 reflections	(Δ/σ)_max_ = 0.001
359 parameters	Δρ_max_ = 0.65 e Å^−^^3^
0 restraints	Δρ_min_ = −0.76 e Å^−^^3^

### Special details

**Table:**

Experimental. Yield, 58%; analysis, calculated for C_40_H_62_Cl_4_N_10_O_16_Cu_4_: C 35.99, H, 4.68; N 10.49%; found: C 35.96, H 4.69, N, 10.51%.
Geometry. All esds (except the esd in the dihedral angle between two l.s. planes) are estimated using the full covariance matrix. The cell esds are taken into account individually in the estimation of esds in distances, angles and torsion angles; correlations between esds in cell parameters are only used when they are defined by crystal symmetry. An approximate (isotropic) treatment of cell esds is used for estimating esds involving l.s. planes.
Refinement. Refinement of F^2^ against ALL reflections. The weighted R-factor wR and goodness of fit S are based on F^2^, conventional R-factors R are based on F, with F set to zero for negative F^2^. The threshold expression of F^2^ > 2sigma(F^2^) is used only for calculating R-factors(gt) etc. and is not relevant to the choice of reflections for refinement. R-factors based on F^2^ are statistically about twice as large as those based on F, and R- factors based on ALL data will be even larger.

### Fractional atomic coordinates and isotropic or equivalent isotropic displacement parameters (Å^2^)

**Table:**

	*x*	*y*	*z*	*U*_iso_*/*U*_eq_	Occ. (<1)
Cu1	0.28269 (4)	0.45069 (3)	0.33563 (4)	0.03039 (18)	
Cu2	0.62990 (4)	0.24708 (3)	0.46234 (4)	0.03051 (18)	
Cl1	0.79310 (12)	0.57100 (8)	0.29681 (13)	0.0599 (4)	
Cl2	0.21665 (12)	0.38831 (7)	0.07826 (11)	0.0475 (3)	
N1	0.4498 (3)	0.45347 (19)	0.3604 (3)	0.0243 (8)	
N2	0.3096 (3)	0.3342 (2)	0.3638 (3)	0.0317 (9)	
N3	0.1119 (3)	0.4486 (2)	0.3195 (3)	0.0315 (9)	
N4	0.7940 (3)	0.2373 (2)	0.4680 (3)	0.0369 (10)	
N5	0.6464 (3)	0.1307 (2)	0.5127 (3)	0.0324 (9)	
O1	0.2790 (3)	0.56694 (18)	0.3491 (3)	0.0464 (9)	
O2	0.3375 (3)	0.69384 (17)	0.3819 (3)	0.0403 (8)	
O3	0.6008 (2)	0.35845 (17)	0.4019 (3)	0.0357 (8)	
O4	0.4616 (3)	0.24340 (16)	0.4152 (3)	0.0345 (8)	
O5	0.2228 (3)	0.4578 (2)	0.1424 (3)	0.0554 (10)	
O6	0.1517 (4)	0.3248 (2)	0.0975 (4)	0.0762 (13)	
O7	0.338 (3)	0.357 (2)	0.123 (4)	0.068 (5)	0.57
O8	0.190 (7)	0.4086 (18)	−0.022 (3)	0.082 (12)	0.57
O7'	0.322 (5)	0.363 (3)	0.081 (9)	0.076 (13)	0.43
O8'	0.143 (7)	0.415 (3)	−0.028 (4)	0.101 (13)	0.43
C1	0.3547 (4)	0.6219 (3)	0.3635 (3)	0.0316 (10)	
C2	0.4679 (4)	0.6027 (2)	0.3540 (3)	0.0281 (10)	
C3	0.5099 (3)	0.5233 (2)	0.3491 (3)	0.0253 (9)	
C4	0.6111 (4)	0.5162 (3)	0.3307 (4)	0.0329 (11)	
H4	0.6391	0.4649	0.3245	0.039*	
C5	0.6689 (4)	0.5847 (3)	0.3218 (4)	0.0373 (11)	
C6	0.6330 (4)	0.6620 (3)	0.3304 (4)	0.0405 (12)	
H6	0.6746	0.7074	0.3256	0.049*	
C7	0.5320 (4)	0.6699 (3)	0.3468 (4)	0.0348 (11)	
H7	0.5061	0.7218	0.3531	0.042*	
C8	0.4971 (4)	0.3808 (2)	0.3830 (3)	0.0274 (10)	
C9	0.4166 (4)	0.3140 (2)	0.3888 (3)	0.0283 (10)	
C10	0.2293 (4)	0.2683 (3)	0.3600 (5)	0.0467 (14)	
H10A	0.2696	0.2291	0.4144	0.056*	
H10B	0.2047	0.2404	0.2939	0.056*	
C11	0.1226 (4)	0.2997 (3)	0.3737 (5)	0.0480 (13)	
H11A	0.0699	0.2546	0.3659	0.058*	
H11B	0.1466	0.3208	0.4435	0.058*	
C12	0.0582 (4)	0.3660 (3)	0.2967 (4)	0.0430 (12)	
H12A	0.0517	0.3496	0.2286	0.052*	
H12B	−0.0201	0.3698	0.2937	0.052*	
C13	0.0402 (4)	0.5036 (3)	0.2348 (4)	0.0489 (14)	
H13A	0.0374	0.4834	0.1700	0.073*	
H13B	0.0738	0.5572	0.2472	0.073*	
H13C	−0.0373	0.5061	0.2322	0.073*	
C14	0.1120 (5)	0.4791 (4)	0.4192 (5)	0.0569 (15)	
H14A	0.1497	0.5313	0.4353	0.085*	
H14B	0.1530	0.4413	0.4738	0.085*	
H14C	0.0332	0.4844	0.4126	0.085*	
C15	0.8213 (5)	0.1484 (3)	0.4798 (5)	0.0523 (14)	
H15A	0.9049	0.1404	0.5088	0.063*	
H15B	0.7881	0.1220	0.4125	0.063*	
C16	0.7717 (4)	0.1115 (3)	0.5501 (5)	0.0500 (14)	
H16A	0.7823	0.0528	0.5524	0.060*	
H16B	0.8127	0.1326	0.6199	0.060*	
C17	0.7999 (6)	0.2666 (4)	0.3708 (6)	0.077 (2)	
H17A	0.7737	0.3221	0.3585	0.115*	
H17B	0.7510	0.2332	0.3139	0.115*	
H17C	0.8787	0.2634	0.3772	0.115*	
C18	0.8777 (4)	0.2825 (4)	0.5567 (5)	0.0631 (17)	
H18A	0.9546	0.2755	0.5593	0.095*	
H18B	0.8753	0.2622	0.6197	0.095*	
H18C	0.8577	0.3393	0.5493	0.095*	
C19	0.5771 (4)	0.0761 (3)	0.4248 (4)	0.0449 (13)	
H19A	0.6019	0.0830	0.3694	0.067*	
H19B	0.4961	0.0897	0.4007	0.067*	
H19C	0.5887	0.0205	0.4478	0.067*	
C20	0.6059 (5)	0.1191 (3)	0.5964 (4)	0.0523 (14)	
H20A	0.6208	0.0640	0.6212	0.078*	
H20B	0.5239	0.1298	0.5700	0.078*	
H20C	0.6467	0.1559	0.6523	0.078*	

### Atomic displacement parameters (Å^2^)

**Table:**

	*U*^11^	*U*^22^	*U*^33^	*U*^12^	*U*^13^	*U*^23^
Cu1	0.0262 (3)	0.0263 (3)	0.0462 (4)	0.0015 (2)	0.0225 (3)	−0.0014 (2)
Cu2	0.0285 (3)	0.0249 (3)	0.0457 (4)	0.0043 (2)	0.0227 (3)	0.0056 (2)
Cl1	0.0542 (9)	0.0542 (8)	0.1007 (12)	0.0039 (7)	0.0617 (8)	0.0139 (8)
Cl2	0.0699 (9)	0.0377 (7)	0.0526 (8)	−0.0002 (6)	0.0430 (7)	0.0007 (6)
N1	0.0249 (19)	0.0217 (18)	0.031 (2)	0.0019 (15)	0.0163 (16)	0.0022 (15)
N2	0.027 (2)	0.0250 (18)	0.050 (2)	−0.0018 (16)	0.0230 (18)	0.0045 (17)
N3	0.0229 (19)	0.038 (2)	0.041 (2)	0.0017 (16)	0.0203 (17)	−0.0030 (17)
N4	0.032 (2)	0.034 (2)	0.054 (3)	0.0090 (17)	0.026 (2)	0.0059 (19)
N5	0.039 (2)	0.0243 (18)	0.036 (2)	0.0013 (17)	0.0172 (18)	0.0004 (16)
O1	0.0350 (19)	0.0281 (17)	0.087 (3)	0.0014 (15)	0.0353 (18)	−0.0093 (17)
O2	0.056 (2)	0.0250 (16)	0.057 (2)	0.0041 (15)	0.0403 (18)	−0.0023 (15)
O3	0.0270 (17)	0.0291 (16)	0.059 (2)	0.0048 (13)	0.0256 (15)	0.0134 (15)
O4	0.0277 (17)	0.0231 (16)	0.055 (2)	0.0021 (13)	0.0191 (15)	0.0086 (14)
O5	0.081 (3)	0.046 (2)	0.051 (2)	0.0051 (19)	0.039 (2)	−0.0035 (17)
O6	0.088 (3)	0.060 (3)	0.100 (4)	−0.019 (2)	0.058 (3)	−0.002 (2)
O7	0.081 (9)	0.068 (7)	0.082 (15)	0.019 (7)	0.061 (11)	0.005 (10)
O8	0.16 (3)	0.049 (8)	0.048 (14)	−0.002 (12)	0.056 (16)	0.000 (7)
O7'	0.074 (16)	0.071 (12)	0.11 (4)	−0.003 (10)	0.07 (2)	−0.012 (19)
O8'	0.13 (3)	0.082 (14)	0.055 (13)	0.019 (15)	−0.002 (14)	0.016 (9)
C1	0.042 (3)	0.030 (2)	0.032 (3)	0.001 (2)	0.025 (2)	0.000 (2)
C2	0.036 (3)	0.030 (2)	0.026 (2)	0.000 (2)	0.020 (2)	−0.0025 (18)
C3	0.029 (2)	0.026 (2)	0.027 (2)	−0.0018 (18)	0.0173 (19)	0.0029 (18)
C4	0.036 (3)	0.030 (2)	0.042 (3)	0.005 (2)	0.025 (2)	0.009 (2)
C5	0.036 (3)	0.043 (3)	0.046 (3)	0.002 (2)	0.030 (2)	0.009 (2)
C6	0.048 (3)	0.035 (3)	0.050 (3)	−0.005 (2)	0.033 (3)	0.006 (2)
C7	0.044 (3)	0.025 (2)	0.046 (3)	−0.001 (2)	0.028 (2)	0.000 (2)
C8	0.029 (2)	0.028 (2)	0.031 (2)	0.0000 (19)	0.019 (2)	0.0015 (18)
C9	0.030 (2)	0.028 (2)	0.036 (3)	0.0021 (19)	0.022 (2)	0.0031 (19)
C10	0.035 (3)	0.037 (3)	0.077 (4)	−0.007 (2)	0.031 (3)	0.006 (3)
C11	0.036 (3)	0.047 (3)	0.072 (4)	−0.006 (2)	0.032 (3)	0.007 (3)
C12	0.024 (2)	0.050 (3)	0.057 (3)	−0.004 (2)	0.019 (2)	−0.003 (3)
C13	0.033 (3)	0.056 (3)	0.065 (4)	0.012 (2)	0.027 (3)	0.015 (3)
C14	0.056 (4)	0.070 (4)	0.058 (4)	0.004 (3)	0.038 (3)	−0.012 (3)
C15	0.043 (3)	0.042 (3)	0.081 (4)	0.009 (2)	0.035 (3)	−0.006 (3)
C16	0.039 (3)	0.035 (3)	0.069 (4)	0.009 (2)	0.015 (3)	0.010 (3)
C17	0.066 (4)	0.095 (5)	0.094 (5)	0.017 (4)	0.059 (4)	0.027 (4)
C18	0.036 (3)	0.055 (3)	0.102 (5)	−0.003 (3)	0.032 (3)	−0.015 (3)
C19	0.049 (3)	0.033 (3)	0.052 (3)	−0.008 (2)	0.020 (3)	−0.004 (2)
C20	0.080 (4)	0.034 (3)	0.058 (4)	0.001 (3)	0.043 (3)	0.007 (2)

### Geometric parameters (Å, °)

**Table:**

Cu1—O1	1.920 (3)	C4—C5	1.373 (6)
Cu1—N2	1.953 (3)	C4—H4	0.9300
Cu1—N1	1.986 (3)	C5—C6	1.368 (6)
Cu1—N3	2.066 (3)	C6—C7	1.385 (7)
Cu1—O5	2.518 (4)	C6—H6	0.9300
Cu2—O4	1.944 (3)	C7—H7	0.9300
Cu2—O3	1.987 (3)	C8—C9	1.516 (6)
Cu2—N5	2.020 (3)	C10—C11	1.521 (7)
Cu2—N4	2.039 (4)	C10—H10A	0.9700
Cu2—O2^i^	2.281 (3)	C10—H10B	0.9700
Cl1—C5	1.750 (5)	C11—C12	1.519 (7)
Cl2—O8	1.36 (4)	C11—H11A	0.9700
Cl2—O7'	1.37 (4)	C11—H11B	0.9700
Cl2—O6	1.417 (4)	C12—H12A	0.9700
Cl2—O5	1.439 (4)	C12—H12B	0.9700
Cl2—O8'	1.47 (4)	C13—H13A	0.9600
Cl2—O7	1.48 (3)	C13—H13B	0.9600
N1—C8	1.313 (5)	C13—H13C	0.9600
N1—C3	1.416 (5)	C14—H14A	0.9600
N2—C9	1.288 (5)	C14—H14B	0.9600
N2—C10	1.467 (5)	C14—H14C	0.9600
N3—C13	1.476 (6)	C15—C16	1.497 (8)
N3—C12	1.490 (6)	C15—H15A	0.9700
N3—C14	1.492 (6)	C15—H15B	0.9700
N4—C18	1.468 (7)	C16—H16A	0.9700
N4—C17	1.482 (7)	C16—H16B	0.9700
N4—C15	1.493 (6)	C17—H17A	0.9600
N5—C20	1.475 (6)	C17—H17B	0.9600
N5—C16	1.478 (6)	C17—H17C	0.9600
N5—C19	1.490 (6)	C18—H18A	0.9600
O1—C1	1.266 (5)	C18—H18B	0.9600
O2—C1	1.247 (5)	C18—H18C	0.9600
O2—Cu2^i^	2.281 (3)	C19—H19A	0.9600
O3—C8	1.275 (5)	C19—H19B	0.9600
O4—C9	1.278 (5)	C19—H19C	0.9600
C1—C2	1.517 (6)	C20—H20A	0.9600
C2—C7	1.394 (6)	C20—H20B	0.9600
C2—C3	1.418 (6)	C20—H20C	0.9600
C3—C4	1.404 (6)		
			
O1—Cu1—N2	164.01 (16)	C5—C6—H6	121.3
O1—Cu1—N1	91.51 (13)	C7—C6—H6	121.3
N2—Cu1—N1	84.45 (13)	C6—C7—C2	122.3 (4)
O1—Cu1—N3	87.77 (13)	C6—C7—H7	118.8
N2—Cu1—N3	95.33 (14)	C2—C7—H7	118.8
N1—Cu1—N3	176.48 (14)	O3—C8—N1	129.5 (4)
O1—Cu1—O5	93.01 (14)	O3—C8—C9	115.4 (4)
N2—Cu1—O5	102.50 (14)	N1—C8—C9	115.1 (4)
N1—Cu1—O5	91.09 (14)	O4—C9—N2	127.1 (4)
N3—Cu1—O5	92.39 (14)	O4—C9—C8	116.4 (4)
O4—Cu2—O3	84.08 (11)	N2—C9—C8	116.4 (4)
O4—Cu2—N5	91.89 (13)	N2—C10—C11	112.0 (4)
O3—Cu2—N5	174.85 (13)	N2—C10—H10A	109.2
O4—Cu2—N4	162.63 (15)	C11—C10—H10A	109.2
O3—Cu2—N4	95.52 (13)	N2—C10—H10B	109.2
N5—Cu2—N4	87.36 (15)	C11—C10—H10B	109.2
O4—Cu2—O2^i^	95.00 (13)	H10A—C10—H10B	107.9
O3—Cu2—O2^i^	87.23 (12)	C12—C11—C10	113.2 (4)
N5—Cu2—O2^i^	96.35 (13)	C12—C11—H11A	108.9
N4—Cu2—O2^i^	102.33 (14)	C10—C11—H11A	108.9
O8—Cl2—O7'	86 (2)	C12—C11—H11B	108.9
O8—Cl2—O6	118 (2)	C10—C11—H11B	108.9
O7'—Cl2—O6	113 (3)	H11A—C11—H11B	107.7
O8—Cl2—O5	112.9 (13)	N3—C12—C11	115.8 (4)
O7'—Cl2—O5	114 (3)	N3—C12—H12A	108.3
O6—Cl2—O5	110.6 (3)	C11—C12—H12A	108.3
O7'—Cl2—O8'	109.0 (19)	N3—C12—H12B	108.3
O6—Cl2—O8'	104 (3)	C11—C12—H12B	108.3
O5—Cl2—O8'	105 (3)	H12A—C12—H12B	107.4
O8—Cl2—O7	107.8 (17)	N3—C13—H13A	109.5
O6—Cl2—O7	103.5 (14)	N3—C13—H13B	109.5
O5—Cl2—O7	102.8 (17)	H13A—C13—H13B	109.5
O8'—Cl2—O7	131 (3)	N3—C13—H13C	109.5
C8—N1—C3	123.6 (4)	H13A—C13—H13C	109.5
C8—N1—Cu1	111.3 (3)	H13B—C13—H13C	109.5
C3—N1—Cu1	125.0 (3)	N3—C14—H14A	109.5
C9—N2—C10	116.6 (4)	N3—C14—H14B	109.5
C9—N2—Cu1	112.5 (3)	H14A—C14—H14B	109.5
C10—N2—Cu1	130.9 (3)	N3—C14—H14C	109.5
C13—N3—C12	107.9 (4)	H14A—C14—H14C	109.5
C13—N3—C14	108.9 (4)	H14B—C14—H14C	109.5
C12—N3—C14	109.6 (4)	N4—C15—C16	109.4 (4)
C13—N3—Cu1	110.2 (3)	N4—C15—H15A	109.8
C12—N3—Cu1	113.5 (3)	C16—C15—H15A	109.8
C14—N3—Cu1	106.8 (3)	N4—C15—H15B	109.8
C18—N4—C17	109.7 (5)	C16—C15—H15B	109.8
C18—N4—C15	110.3 (4)	H15A—C15—H15B	108.3
C17—N4—C15	109.0 (4)	N5—C16—C15	110.3 (4)
C18—N4—Cu2	110.8 (3)	N5—C16—H16A	109.6
C17—N4—Cu2	111.6 (3)	C15—C16—H16A	109.6
C15—N4—Cu2	105.3 (3)	N5—C16—H16B	109.6
C20—N5—C16	110.6 (4)	C15—C16—H16B	109.6
C20—N5—C19	108.2 (4)	H16A—C16—H16B	108.1
C16—N5—C19	110.3 (4)	N4—C17—H17A	109.5
C20—N5—Cu2	112.6 (3)	N4—C17—H17B	109.5
C16—N5—Cu2	105.6 (3)	H17A—C17—H17B	109.5
C19—N5—Cu2	109.5 (3)	N4—C17—H17C	109.5
C1—O1—Cu1	132.6 (3)	H17A—C17—H17C	109.5
C1—O2—Cu2^i^	128.7 (3)	H17B—C17—H17C	109.5
C8—O3—Cu2	110.4 (3)	N4—C18—H18A	109.5
C9—O4—Cu2	111.3 (3)	N4—C18—H18B	109.5
Cl2—O5—Cu1	124.0 (2)	H18A—C18—H18B	109.5
O2—C1—O1	121.7 (4)	N4—C18—H18C	109.5
O2—C1—C2	117.6 (4)	H18A—C18—H18C	109.5
O1—C1—C2	120.6 (4)	H18B—C18—H18C	109.5
C7—C2—C3	119.1 (4)	N5—C19—H19A	109.5
C7—C2—C1	115.7 (4)	N5—C19—H19B	109.5
C3—C2—C1	125.2 (4)	H19A—C19—H19B	109.5
C4—C3—N1	121.3 (4)	N5—C19—H19C	109.5
C4—C3—C2	117.9 (4)	H19A—C19—H19C	109.5
N1—C3—C2	120.8 (4)	H19B—C19—H19C	109.5
C5—C4—C3	120.2 (4)	N5—C20—H20A	109.5
C5—C4—H4	119.9	N5—C20—H20B	109.5
C3—C4—H4	119.9	H20A—C20—H20B	109.5
C6—C5—C4	123.0 (4)	N5—C20—H20C	109.5
C6—C5—Cl1	119.5 (4)	H20A—C20—H20C	109.5
C4—C5—Cl1	117.5 (4)	H20B—C20—H20C	109.5
C5—C6—C7	117.4 (4)		

Symmetry codes: (i) −*x*+1, −*y*+1, −*z*+1.

### Hydrogen-bond geometry (Å, °)

**Table:**

*D*—H···*A*	*D*—H	H···*A*	*D*···*A*	*D*—H···*A*
C6—H6···O7^ii^	0.93	2.58	3.26 (3)	129
C16—H16A···O8^iii^	0.97	2.46	3.41 (3)	168
C20—H20C···O2^i^	0.96	2.54	3.139 (6)	121
C4—H4···O3	0.93	2.21	2.799 (5)	120
C7—H7···O2	0.93	2.36	2.714 (6)	102
C13—H13A···O5	0.96	2.55	3.158 (7)	121
C13—H13B···O1	0.96	2.39	2.960 (6)	118
C14—H14A···O1	0.96	2.46	3.026 (7)	117
C17—H17A···O3	0.96	2.56	3.103 (7)	116
C19—H19B···O4	0.96	2.58	3.084 (6)	113

Symmetry codes: (ii) −*x*+1, *y*+1/2, −*z*+1/2; (iii) −*x*+1, *y*−1/2, −*z*+1/2; (i) −*x*+1, −*y*+1, −*z*+1.

## References

[bb1] Bruker, (1998). *SMART* and *SAINT* Bruker AXS Inc., Madison, Wisconsin, USA.

[bb2] Sheldrick, G. M. (1996). *SADABS* University of Göttingen, Germany.

[bb3] Sheldrick, G. M. (2008). *Acta Cryst.* A**64**, 112–122. 10.1107/S0108767307043930 18156677

[bb4] Spek, A. L. (2009). *Acta Cryst.* D**65**, 148–155. 10.1107/S090744490804362X PMC2631630 19171970

[bb5] Tao, R. J., Zang, S. Q., Cheng, Y. X., Wang, Q. L., Hu, N. H., Niu, J. Y. & Liao, D. Z. (2003). *Polyhedron*, **22**, 2911–2916.

[bb6] Zang, S. Q., Tao, R. J., Wang, Q. L., Hu, N. H., Cheng, Y. X., Niu, J. Y. & Liao, D. Z. (2003). *Inorg. Chem.* **42**, 761–766. 10.1021/ic020491j 12562190

